# Metformin alleviates muscle wasting post-thermal injury by increasing Pax7-positive muscle progenitor cells

**DOI:** 10.1186/s13287-019-1480-x

**Published:** 2020-01-08

**Authors:** Yusef Yousuf, Andrea Datu, Ben Barnes, Saeid Amini-Nik, Marc G. Jeschke

**Affiliations:** 1 Sunnybrook Research Institute, 2075 Bayview Ave., Rm. D704, Toronto, ON M4N 3M5 Canada; 2 Laboratory in Medicine and Pathobiology, University of Toronto, Toronto, Canada; 3 Division of Plastic Surgery, Department of Surgery, University of Toronto, Toronto, Canada; 4 Ross Tilley Burn Centre, Sunnybrook Health Sciences Centre, Toronto, Canada; 5 Department of Immunology, University of Toronto, Toronto, Canada

**Keywords:** Metformin, Skeletal muscle, Muscle wasting, Thermal injury, Burn, Satellite cells, Pax7, Fat infiltration

## Abstract

**Background:**

Profound skeletal muscle wasting and weakness is common after severe burn and persists for years after injury contributing to morbidity and mortality of burn patients. Currently, no ideal treatment exists to inhibit muscle catabolism. Metformin is an anti-diabetic agent that manages hyperglycemia but has also been shown to have a beneficial effect on stem cells after injury. We hypothesize that metformin administration will increase protein synthesis in the skeletal muscle by increasing the proliferation of muscle progenitor cells, thus mitigating muscle atrophy post-burn injury.

**Methods:**

To determine whether metformin can attenuate muscle catabolism following burn injury, we utilized a 30% total burn surface area (TBSA) full-thickness scald burn in mice and compared burn injuries with and without metformin treatment. We examined the gastrocnemius muscle at 7 and 14 days post-burn injury.

**Results:**

At 7 days, burn injury significantly reduced myofiber cross-sectional area (CSA) compared to sham, *p* < 0.05. Metformin treatment significantly attenuated muscle catabolism and preserved muscle CSA at the sham size. To investigate metformin’s effect on satellite cells (muscle progenitors), we examined changes in Pax7, a transcription factor regulating the proliferation of muscle progenitors. Burned animals treated with metformin had a significant increase in Pax7 protein level and the number of Pax7-positive cells at 7 days post-burn, *p* < 0.05. Moreover, through BrdU proliferation assay, we show that metformin treatment increased the proliferation of satellite cells at 7 days post-burn injury, *p* < 0.05.

**Conclusion:**

In summary, metformin’s various metabolic effects and its modulation of stem cells make it an attractive alternative to mitigate burn-induced muscle wasting while also managing hyperglycemia.

## Introduction

Burn injury results in a debilitating stress response termed the hypermetabolic response resulting in profound changes to several organ systems. Despite recent advances in therapeutic strategies such as protocolized acute burn care, enhanced wound coverage, improved resuscitation, and suitable infection control, severe burns still affect nearly every organ system resulting in significant morbidity and mortality [1–6]. A significant increase in circulating catecholamines, glucocorticoids, glucagon, and dopamine secretion is thought to initiate the cascade of events leading to this hypermetabolic response [7–9]. A hallmark of the hypermetabolic response is significant muscle wasting, weakness, and debilitation, which persists for the duration of the hypermetabolic response [10, 11]. This muscle wasting occurs in muscles distal to the burn site and is due to proteolysis to provide proteins and amino acids for the hugely increased metabolic demands. While this process is per se needed, the ensuing catabolism and associated weakness complicate and delays recovery. Several groups are trying to identify novel treatment approaches to mitigate this catabolism and hypothesize that a reduced catabolic response would improve outcomes after burn. Although investigations to date have identified probable leads and developed useful strategies to manage inflammation and muscle cachexia, no satisfactory drugs are yet available to curtail these conditions.

Metformin is an anti-diabetic agent that is recommended as a first-line oral therapy for type 2 diabetes (T2D) [12]. Metformin manages hyperglycemia by decreasing hepatic glucose production through the inhibition of mitochondrial respiratory-chain complex 1 [13]. With regard to skeletal muscle, metformin increases glucose uptake through upregulation of the glucose transporter type 4 (GLUT4) [14–17]. In addition to these effects, metformin also activates the cellular energy sensor, AMP-activated protein kinase (AMPK) [17], which has a wide range of effects throughout the body in numerous organs which will be discussed in more detail later. The activation of AMPK leads to the inhibition of hepatic gluconeogenesis and peripheral glucose uptake in the skeletal muscle [17]. This activation of AMPK leads to the translocation of GLUT4 and increased glucose uptake and glycolysis within the skeletal muscle. Under conditions of severe burn injury, adenylate cyclase converts ATP to AMP, reducing levels of ATP and increasing AMP [18, 19]. This conversion reaches its highest peak at 72 h post-burn injury and results in activation of AMPK and phosphorylation of mTOR which initiates autophagy pathways [18, 19].

The therapeutic potential of metformin is not just limited to its ability to manage hyperglycemia and diabetes. Recently, metformin has been shown to effectively treat several diseases including cancer [20–22], cardiovascular diseases [23], and brain trauma [24, 25]. Moreover, there is evidence to suggest that metformin’s pleiotropic effects delay the aging process [26, 27]. Several studies have shown that metformin can rescue muscle wasting in response to cardiovascular injury or skeletal muscle injury caused by cardiotoxin [28–31]. Recently, metformin has been found to promote the differentiation of human and mouse neural stem cells in culture. Moreover, after brain injury, metformin treatment increases the proliferation of endogenous neural stem cells, increases their total number of neural stem cells, and improves sensory-motor function after brain injury in mice [24]. Considering metformin’s beneficial effects on neural stem cells in the context of injury, it is plausible that metformin has a similarly beneficial effect on muscle progenitor cells.

Skeletal muscle regeneration is dependent on contribution from muscle resident stem cells, named satellite cells. Satellite cells are marked by the paired-box transcription factor 7 (Pax7). Satellite cells are essential for skeletal muscle regeneration following injury [32] and for muscle hypertrophy and homeostasis [33–35]. Recent reports have illustrated a reduction in satellite cell numbers and an increase in myonuclear apoptosis post-burn injury in both humans and mice [36, 37]. Dysregulation of satellite cells may impair their ability to repair skeletal muscle after thermal injury. Indeed, depletion of satellite cells worsens muscle catabolism in mice after scald-burn injury [38] illustrating the importance of satellite cells in the recovery of lean muscle mass. Metformin is a drug that could potentially target satellite cells to prevent their dysregulation after thermal injury leading to less erosion of muscle mass. Gore et al. demonstrated that metformin treatment increases protein synthesis in severe burn patients [39]. One explanation for this increase in protein synthesis may be metformin increasing the proliferation of muscle progenitor cells (satellite cells). These findings suggest the diverse effects of metformin may extend into treating burn patients and improving their outcomes. Insulin resistance and muscle wasting are chronic complications of burn trauma. Metformin targets both insulin resistance and muscle wasting and is economically beneficial and easily administered orally. These advantages make it an attractive alternative for the long-term treatment of burn patients.

To date, the effects of metformin on muscle proteolysis and structure are essentially unknown. Using a 30% total burn surface area (TBSA) murine burn model, we examined the effect of metformin treatment on mitigating burn-induced muscle wasting. We hypothesized that metformin treatment would (1) increase the proliferation of satellite cells after severe burn injury in the gastrocnemius muscle distal from the burn site and (2) attenuate muscle wasting after severe burn injury.

## Materials and methods

### Mice

All mice used were male, 8 weeks old, and C57BL/6. Mice were randomly divided into the following groups: sham, burn, and burn + metformin treatment (*n* = 18 per group). Within each group, mice were subdivided into groups sacrificed at three different time points: 2 days, 7 days, and 14 days post-thermal injury (*n* = 6 per group). The animal experiments were performed in accordance with the guidelines and regulations set forth by the Sunnybrook Research Institute and Sunnybrook Health Sciences Animal Policy and Welfare Committee of the University of Toronto, Ontario Canada. The Sunnybrook animal care committee approved all animal experiments (approval #15-503(M-1)) under the auspices of the Canadian Council on Animal Care.

### Burn

Animals were anesthetized with isoflurane and received an intraperitoneal (IP) buprenorphine injection (0.1 mg/kg). The dorsum of the animal was shaved, and lactated Ringer’s solution was subcutaneously injected along the spine. The mice were placed in a mold that exposes the dorsum to a pre-determined TBSA. A 30% TBSA full-thickness scald injury was induced by exposing the dorsum of the animal to water pre-heated to 98 °C for 10 s and the ventrum for 2 s. Following the burn, the animals were placed in separate cages. Sham animals were anesthetized and received buprenorphine injection but did not receive a thermal injury.

### Metformin treatment and cell proliferation analysis

Each animal in the metformin group was injected intraperitoneally with 100 mg/kg of metformin hydrochloride (Sigma-Aldrich) dissolved in 1× PBS. Injections began 24 h after burn injury and continued every day at the same time until endpoint. To analyze cell proliferation in the skeletal muscle, we injected animals with 5-Bromo-2′-deoxyuridine (BrdU) (Sigma) 24 h prior to harvest. Each animal received an intraperitoneal injection with 250 μL of 2 mg/ml BrdU.

### Muscle harvest and dry/wet muscle ratio

We dissected the gastrocnemius muscle from mice 2, 7, and 14 days after exposure to cutaneous thermal injury for histological and protein analysis. Whole gastrocnemius muscle was also weighed at the time of harvest to obtain the wet muscle weight. The dry muscle weight was obtained by dehydrating the whole gastrocnemius muscle for 5 days at 50 °C. The dry muscle weight was weighed. The dry muscle weight was divided by the wet muscle weight to obtain the dry/wet muscle ratio. For histology, muscle samples were tied to a support prior to excision to prevent contraction. Samples were either snap frozen in liquid nitrogen-cooled isopentane or fixed in 10% neutral buffered formalin for 24 h.

### Hematoxylin and eosin staining (H&E)

Frozen sections were allowed to dry for 5 min at room temperature. Sections were then stained with Mayer’s hematoxylin (Sigma-Aldrich) for 10 min and rinsed in running tap water. Sections were then dipped in 0.5% eosin 12 times and dipped in distilled water until eosin stops streaking. Sections were then dehydrated in various ethanol solutions and xylene. Finally, slides were mounted and cover slipped with xylene-based aqueous mounting media (SHUR/Mount™).

### Myofiber cross-sectional area analysis

Representative images of the gastrocnemius muscle sections were captured at × 20 magnification. The cross-sectional area of individual myofiber was obtained through ImageJ® software. For each animal, the cross-sectional areas of approximately 500 myofibers were counted blindly in five images (field-of-views) and subsequently the average cross-sectional area was determined. We did not differentiate between type I and type II fibers when measuring muscle cross-sectional area.

### Western blot

The gastrocnemius muscle was harvested, and protein was isolated from tissue lysates using RIPA lysis buffer. Protein concentrations were then measured using bicinchoninic acid (BCA) assay as previously reported [40]. Briefly, 30 mg of each protein sample was separated by SDS-polyacrylamide gel electrophoresis, transferred to a nitrocellulose membrane, blocked with 5% milk in tris-buffered saline/0.1% Tween 20, and hybridized with the following primary antibodies: anti-Pax7 (1:500, DHSB), anti-AMPKα (1:1000, Cell Signaling), anti-Phospho-AMPKα (Thr172) (1:1000, Cell Signaling), and GAPDH (1:5000, Cell Signaling). The membranes were then incubated with anti-rabbit or anti-mouse horseradish peroxidase (HRP)-conjugated secondary antibody (1:2500, Santa Cruz). Detection of the signal was accomplished using western HRP chemiluminescence (ECL) reagents (Bio-Rad Laboratories), and imaging of the blots was performed using ChemiDoc™ MP System (Bio-Rad). To analyze the blots, Image Lab™ Software (Bio-Rad) was used to quantify band intensity and calculate the absorbance ratio of the target protein to the loading control, GAPDH.

### Immunohistochemistry

Gastrocnemius muscle samples for histological analysis were collected and fixed in 10% formalin for 24 h and transferred to 70% ethanol. Samples were then embedded in paraffin and sectioned at 5 μm across the transverse plane. Paraffin-embedded slides were heated at 60 °C for 30 min and deparaffinized with citrosol and rehydrated through a series of decreasing alcohol concentrations. Antigen decloaker solution (Biocare Medical) was preheated in an antigen decloaking chamber at 70 °C for 20 min before slides were added. The slides were then heated at 100 °C in the antigen decloaking solution for 4 min, cooled to 60 °C, and washed with tap water. After blocking endogenous peroxidase activity with 3% H_2_O_2_ for 10 min, sections were incubated with the following primary antibodies: anti-Pax7 (1:100, DHSB), anti-MPO (1:200, Abcam), and anti-NF-κB p65 (1:200, Cell Signaling). Slides were washed with washing buffer (0.05 M Tris-HCl, 0.15 M NaCl, and 0.05% Tween 20 in double distilled water). Sections were then incubated in MACH3 probe (Biocare Medical) for 15 min and washed, and MACH3 horseradish peroxidase polymer detection was added for 15 min. After washing again, betazoid diaminobenzidine (DAB) chromogen kits (Biocare Medical) were mixed and incubated for 10 min, or until the brown color was observed. Slides were rinsed in running tap water, stained with hematoxylin for 30 s, washed, and differentiated in 1.5% acid alcohol briefly. Slides were then placed in 0.1% sodium bicarbonate for 10 s and dehydrated in citrosol and alcohol solutions. Lastly, slides were mounted and cover slipped with xylene-based aqueous mounting media (SHUR/Mount™).

For quantification, five different fields were randomly chosen for each sample. The sections were imaged via an optical microscope (Leica Microsystems) with × 20 and × 40 objective lenses. The percentage of positive cells for each target was determined by dividing the number of positive cells by the total number of nuclei in each histological field. The average ratio for each subject was considered. Negative controls without primary antibody but with DAB staining were prepared to confirm the staining observed.

### Immunofluorescence

Frozen muscle samples were embedded in OCT and frozen in liquid nitrogen-cooled isopentane. The samples were cut perpendicularly via a cryostat (10-μm thickness). Sections were allowed to cool for 5 min at room temperature and subsequently fixed in 4% paraformaldehyde (PFA) for 5 min. Sections were washed with PBS and incubated with glycine solution to quench the PFA signal. For BrdU staining, sections were incubated in 1.5 M HCl for 30 min at 37 °C and subsequently neutralized in 0.1 M borate buffer solutions for 5 min. After washing, sections were permeabilized in 0.25% Triton-X for 10 min and washed again. Sections were then incubated in blocking buffer (5% normal goat serum, 2% BSA, and mouse-on-mouse blocking reagent diluted PBS) for 1 h at room temperature. Sections were rinsed in PBS and incubated in primary antibody overnight at 4 °C overnight. The following antibodies were used: anti-Pax7 (1:100, mouse, DHSB), anti-BrdU (1:250, rat, Abcam), and Laminin (1:200, rabbit, Abcam). Sections were rinsed with PBS and incubated in secondary antibody solution diluted in blocking buffer: goat anti-rabbit Alexa Fluor 488 (1:1000), goat anti-mouse IgG1 Alexa Fluor 546 (1:1000), or goat anti-mouse IgG1 Alexa Fluor 488 (1:1000). Sections were rinsed and mounted with fluorescent mounting media containing DAPI (Vector Laboratories). Samples were imaged with a Zeiss Apotome fluorescent microscope.

### Oil Red O staining

The gastrocnemius muscle was harvested, snap frozen in liquid nitrogen-cooled isopentane, and embedded in OCT. Using a cryostat, the muscle was cut perpendicularly (thickness 10 μm) and added onto the slide. Slides were fixed in formalin and briefly washed with running tap water. Sections were then rinsed in 60% isopropanol and stained with freshly prepared Oil Red O working solution for 15 min. After another rinse with 60% isopropanol, nuclei were lightly stained with hematoxylin (Sigma-Aldrich) for 30 s and rinsed with distilled water. Slides were mounted and cover slipped with xylene-based aqueous mounting media (SHUR/Mount™).

### Statistical analysis

Statistical analysis was performed using one-way ANOVA. Data are represented as mean ± SEM (*n* = 6). *p* < 0.05 were taken as statistically significant.

## Results

### Metformin treatment attenuates muscle wasting in mice

**Fig. 1:**
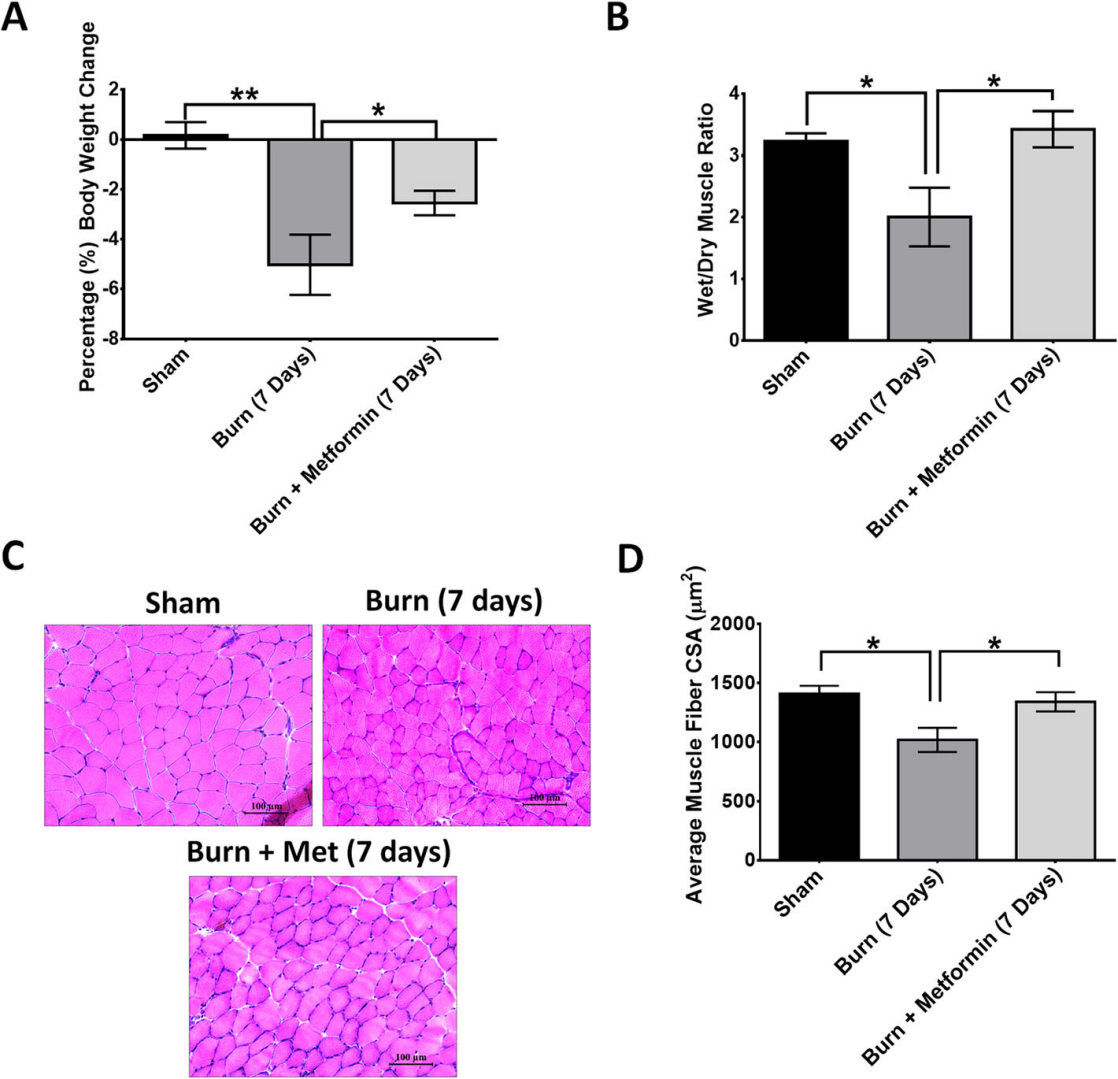
Metformin treatment attenuates muscle wasting in severely burned mice. **a** The percentage (%) body weight change in sham, burn, and burn + metformin at 7 days post-burn in mice. **b** Gastrocnemius muscle mass expressed as a dry/wet muscle ratio in sham, burn, and burn + metformin at 7 days post-burn in mice. **c** Representative images of hematoxylin and eosin staining of the gastrocnemius muscle from sham, burn, and burn + metformin mice at 2 and 7 days post-burn injury. Myofibers are significantly smaller at 7 days post-burn injury. Metformin treatment restores myofiber size. Images were taken at × 20 magnification. **d** Quantification of muscle cross-sectional area (μm^2^) in sham, burn, and burn + metformin at 2 and 7 days post-burn injury

To assess whether metformin attenuates muscle catabolism after severe burn injury, we examined animal weights, the dry/wet muscle ratio, and the cross-sectional muscle area. As expected, animal weights in the burn groups decreased significantly at 7 days, and this was sustained until 14 days post-thermal injury (Fig. 1a), *p* < 0.05. This corresponded to a 5% decrease in weight. In contrast, with metformin treatment, there was only a 2.5% decrease in weights, a difference that is significant compared to the burn groups (Fig. 1a), *p* < 0.05. This indicates that metformin attenuates muscle catabolism post-thermal injury. Lastly, the change in muscle weight relative to body weight (muscle weight/body weight) can be seen in Additional file 1.

To examine changes in muscle histology, we performed hematoxylin and eosin (H&E) staining. There was a significant reduction in myofiber cross-sectional area at these 7 days in the burn group (Fig. 1c, d), *p* < 0.05. Several studies have shown that metformin can rescue muscle wasting in response to cardiovascular injury or skeletal muscle injury caused by cardiotoxin [28–31]. Indeed, in our burn-induced muscle wasting model, metformin treatment attenuated muscle wasting at 7 days post-thermal injury (Fig. 1c, d), *p* < 0.05. There was no significant difference in dry/wet muscle ratio and muscle cross-sectional between the metformin-treated group and the sham group at 7 days post-thermal injury (Fig. 1b). These results suggest that metformin mitigates muscle wasting in severely burned mice.

### Metformin does not affect myofiber size at 14 days

**Fig. 2:**
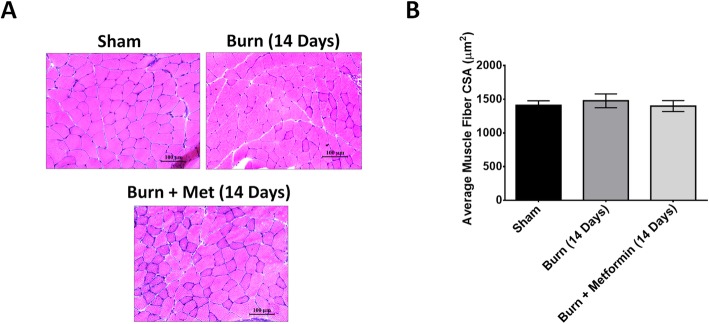
Metformin has no effect on myofiber size at 14 days. **a** Representative images of hematoxylin and eosin staining of the gastrocnemius muscle from sham, burn, and burn + metformin mice at 14 days post-burn injury. Images were taken at × 20 magnification. **b** Quantification of muscle cross-sectional area (μm^2^) in sham, burn, and burn + metformin at 14 days post-burn injury

At 14 days post-thermal injury, we observed no differences in the dry/wet muscle ratio or the muscle cross-sectional area between sham, burn, and metformin groups (Fig. 2a, b). We hypothesize that by day 14 post-thermal injury, the mice have likely recovered in terms of their lean muscle mass regardless of metformin treatment. This might be expected due to the reduced morbidity observed in mice as a result of accelerated healing time [41] and differences in immune function [42] compared to humans. Moreover, as opposed to humans, mice are highly mobile after burn injury which may accelerate recovery of muscle mass. Metformin seems to exert its effect on the skeletal muscle and myofiber size early in the burn response of mice.

### Metformin treatment reduces fat infiltration in the skeletal muscle

**Fig. 3:**
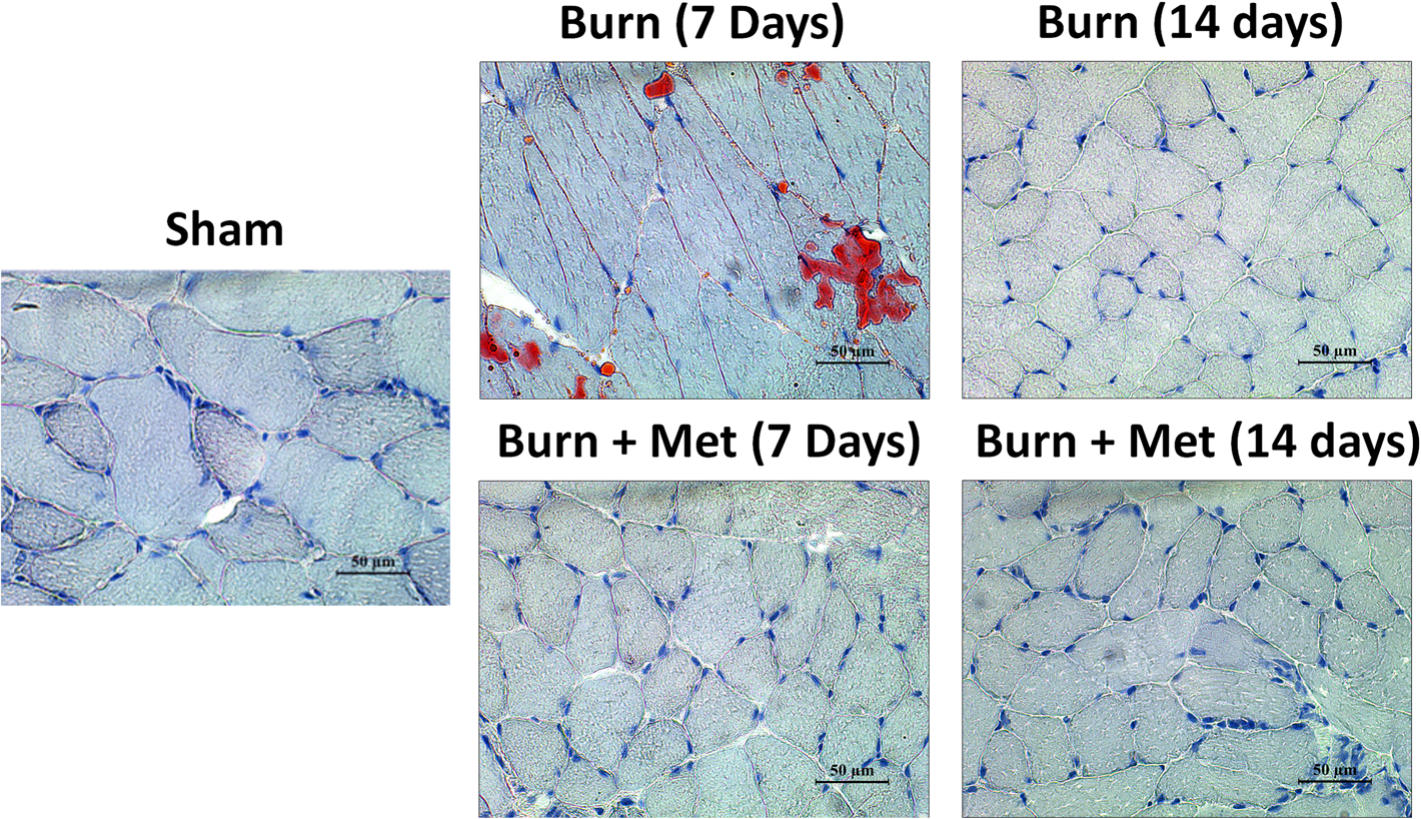
Metformin reduces fat infiltration in the skeletal muscle after severe burn injury. Representative Oil Red O images. Images were taken at × 40 magnification. Metformin treatment reduced fat infiltration in mice at 7 days post-burn injury

After burn injury, there is massive lipolysis and release of free fatty acids that lead to fat infiltration in several organs, including the liver and skeletal muscle [43]. Intramuscular fat accumulation in the skeletal muscle is linked with decreased muscle strength, reduced insulin sensitivity, and increased mortality [44]. Metformin has numerous systemic effects, one of which is reducing fat infiltration in the liver [45–47] and kidney [48]. Therefore, metformin may have indirect effects on the skeletal muscle such as reducing fat infiltration that facilitates recovery of lean muscle mass. To examine fat infiltration in the muscle, we performed Oil Red O staining to visualize intramuscular lipid droplets. There was fat infiltration at 7 days post-thermal injury in the burn group when compared with sham and metformin animals (Fig. 3). With metformin treatment, we observed less fat infiltration compared to the burn group (Fig. 3). The extent of Oil Red O staining in the metformin group was comparable to the sham group (Fig. 3).

### Metformin treatment activates AMPKα in the skeletal muscle after severe burn injury

**Fig. 4:**
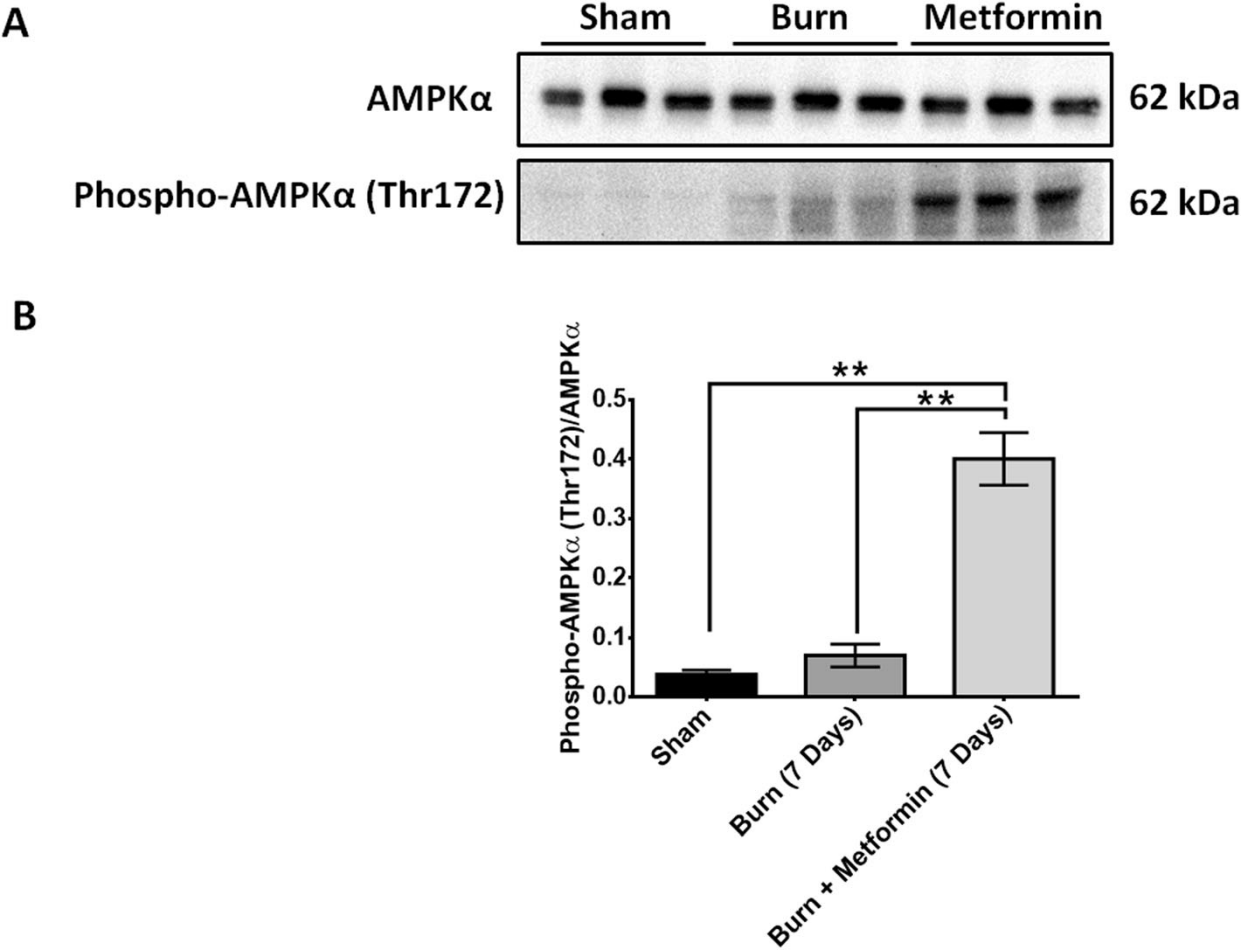
Metformin treatment activates AMPKα in the skeletal muscle after severe burn injury. **a** Representative western blot for AMPKα and phospho-AMPKα (Thr172). **b** Quantification of phospho-AMPKα (Thr172) protein level normalized to AMPKα

To evaluate pathway activation of metformin in our burn model, we performed western blotting for AMPKα and the activated version of AMPK, phospho-AMPKα (Thr172). We observed no significant difference in the protein level of AMPKα between the sham, burn-, and metformin-treated groups at 7 days (Fig. 4a, b). However, there was a significant increase in the protein level of phospho-AMPKα in the metformin treatment group at 7 days post-thermal injury (Fig. 4a, b), *p* < 0.05. We did not observe an increase in phospho-AMPKα in the burn group. This might be because 7 days post-burn is too late of a time point to examine as activation of AMPK occurs during the acute phase of the burn response (i.e., 72 h or less) [18, 19]. Nevertheless, these results confirm that metformin-activated AMPK and AMPKα phosphorylation may be the mediator by which metformin alleviated muscle catabolism. Studies suggest that chronic activation of AMPKα in the skeletal muscle increases the expression of muscle hexokinase and glucose transporter 4 (GLUT4), which mimics the effects of exercise training in muscle [49].

### Metformin treatment increases the number of Pax7+ cells and protein level at 7 days in the skeletal muscle after severe burn injury

**Fig. 5:**
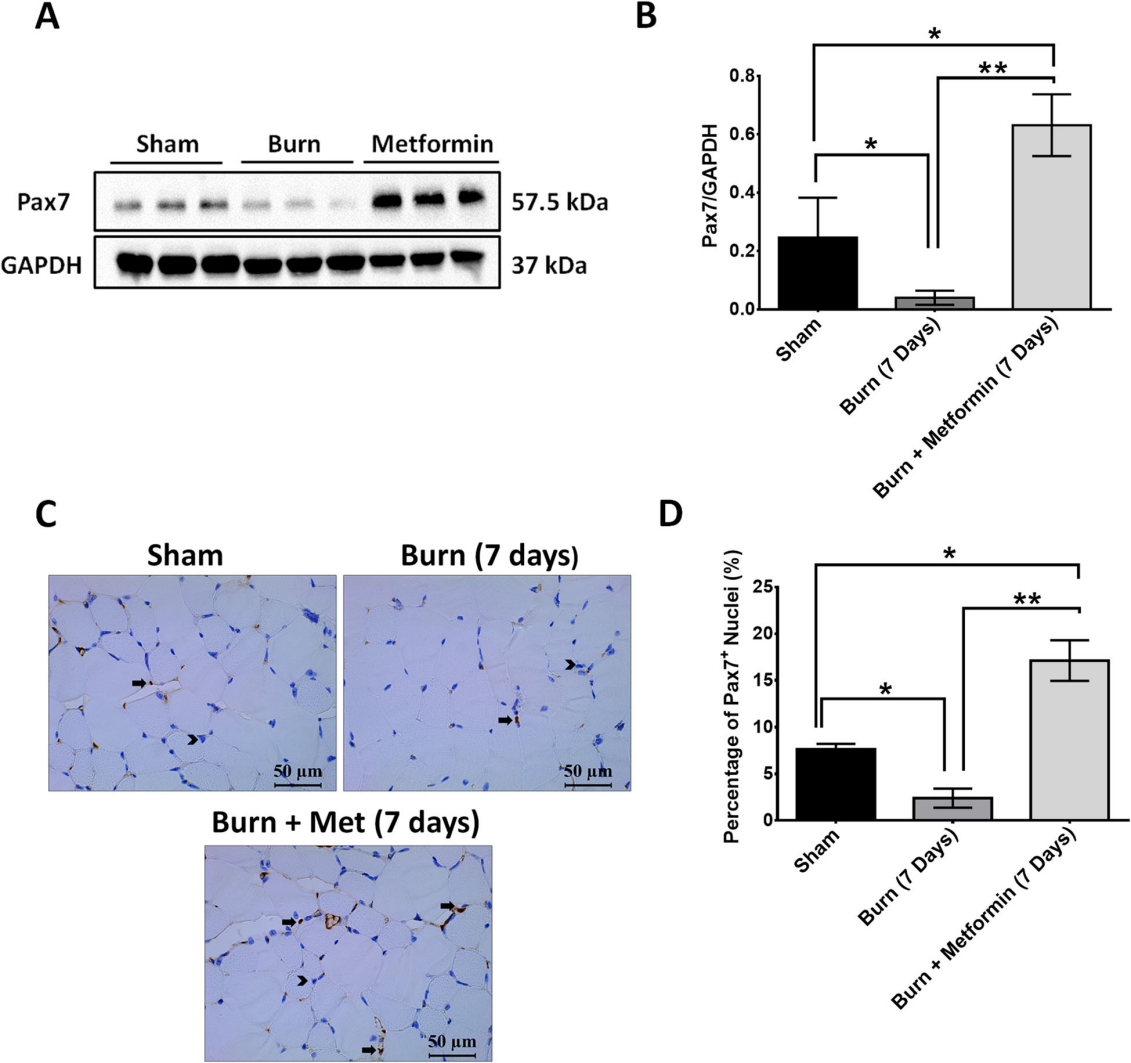
Metformin treatment increases the number of Pax7+ cells and protein level at 7 days in the skeletal muscle after severe burn injury. **a** Representative western blot for Pax7 and GAPDH protein. **b** Quantification of Pax7 protein level normalized to GAPDH. **c** Representative images of Pax7 immunohistochemistry in sham, burn, and burn + metformin mice at 7 days post-burn injury. Arrows indicate positive cells, and arrowheads show negative cells. Images were obtained at × 40 magnification. **d** Quantification of Pax7-positive nuclei in sham, burn, and burn + metformin mice at 7 days post-burn injury

The importance of AMPKα activation in attenuating burn-induced muscle wasting is further supported by the fact that AMPKα activation in satellite cells is essential for muscle regeneration [50]. To examine whether metformin treatment influences satellite cell activity after severe burn injury, we performed immunohistochemistry and western blotting for Pax7, a transcription factor expressed by quiescent and proliferating satellite cells. Protein expression of Pax7 in the burn group was significantly reduced in the muscle at 7 days post-thermal injury when compared with sham and metformin groups, which is in line with our previous report (Fig. 5a, b) [37]. Interestingly, metformin significantly increased protein expression of Pax7 compared to sham and burn groups (Fig. 5a, b), *p* < 0.05. Immunohistochemistry revealed a significant reduction in the number of Pax7-positive nuclei in the burn group compared to sham and metformin-treated groups at 7 days post-thermal injury (Fig. 5c, d), *p* < 0.05. Metformin treatment significantly increased the number of Pax7-positive nuclei compared to sham and burn groups (Fig. 5c, d), *p* < 0.05. This increase in the number of satellite cells may have contributed to the rescue of muscle wasting observed in metformin-treated mice. Another possibility is that metformin enhanced anabolism of the skeletal muscle at an earlier time point post-burn resulting in the recovery of Pax7+ cells earlier. A greater pool of satellite cells (muscle progenitors) increases the regenerative capacity of the skeletal muscle to build new myofibers.

### Metformin treatment increases the proliferation of satellite cells in the skeletal muscle

**Fig. 6:**
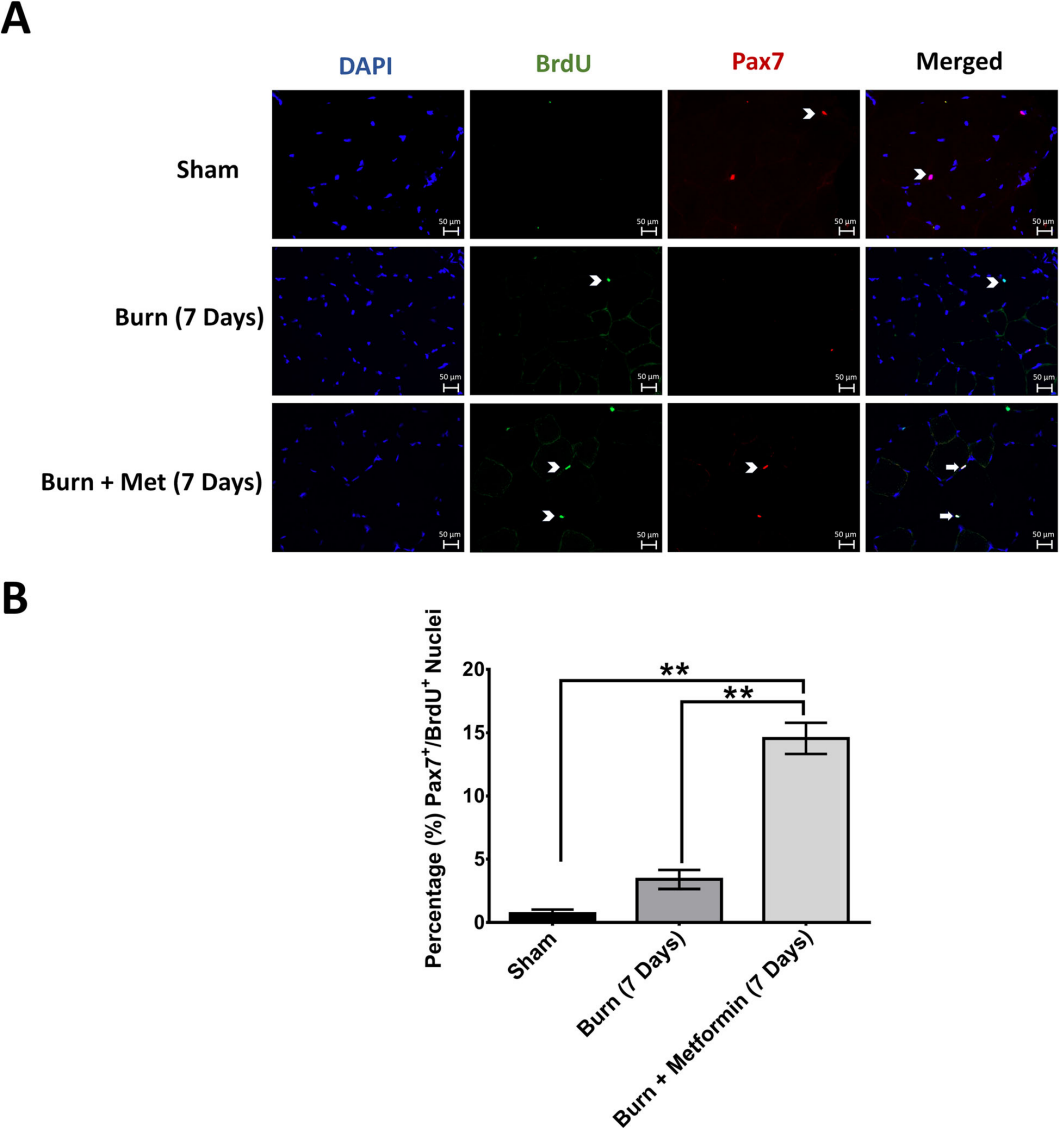
Metformin treatment increases the proliferation of satellite cells in the skeletal muscle. **a** Representative double immunofluorescence staining showing that Pax7^+^ cells were also positive for BrdU at 7 days post-burn injury. Images were taken at × 40 magnification. Arrowheads indicate single-positive cells, and arrows show double-positive cells. **b** Quantification of Pax7^+^ cells that were also BrdU positive at 7 days post-burn injury

To further assess whether metformin affects satellite cells or the changes in satellite cells are secondary to the effect of metformin on muscles after severe burn injury, we investigated metformin’s effect on the proliferation of these stem cells. The increase in Pax7-positive cells observed in the metformin group at 7 days may be due to increased proliferation caused by metformin. Recently, metformin has been shown to increase the absolute number of neural precursor cells in mice and increase their proliferation in response to brain injury [24, 25]. To determine whether metformin has a similar effect in satellite cells, we performed immunofluorescence double staining for Pax7 and BrdU to identify muscle progenitors that were proliferating. Animals were injected with BrdU 24 h prior to sacrifice to label proliferating myonuclei. Quantification of the proportion of Pax7/BrdU-positive cells revealed a significant increase in proliferating Pax7-positive cells as well as a total number of Pax7-positive cells (Fig. 6a, b), *p* < 0.05 (Additional file 2). Fifteen percent of Pax7-positive cells were positive for BrdU in the metformin group indicating a proliferation rate of 15% after burn injury (Fig. 6b). Collectively, these data suggest that metformin increases the proliferation and the total number of muscle progenitor cells in the skeletal muscle after severe burn injury. Metformin’s mitigation of muscle wasting after burn injury may in part be due to its beneficial effects on muscle progenitors and the regenerative capacity of the skeletal muscle.

### Metformin treatment does not attenuate inflammation in the skeletal muscle of severely burned mice

**Fig. 7:**
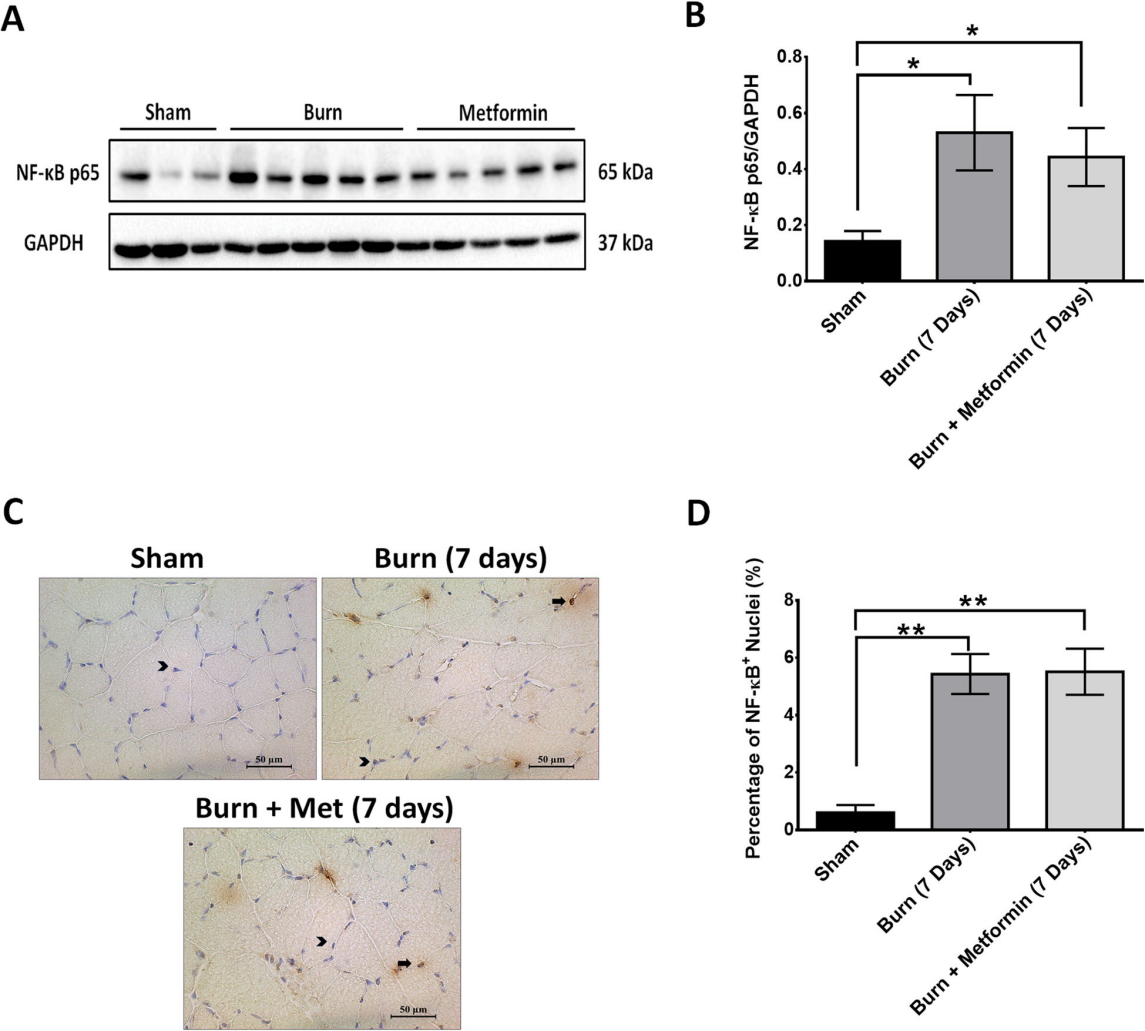
Metformin treatment does not attenuate inflammation in the skeletal muscle of severely burned mice. **a** Representative western blot for NF-κB. **b** Quantification of NF-κB protein expression normalized to GAPDH. **c** Representative images of NF-κB immunohistochemistry. Images were obtained at × 40 magnification. Arrows indicate NF-κB-positive nuclei, and arrowheads indicate NF-κB-negative nuclei. **d** Quantification of NF-κB-positive nuclei in sham, 7 days, and 7 days + metformin post-burn

We have recently shown that the reduction in satellite cells post-burn injury is tightly associated with an inflammatory cascade. Metformin has anti-inflammatory properties [51]. Studies have suggested that metformin suppresses inflammation in diabetes and intestinal inflammation by inhibiting the activity of NF-κB via AMPK-independent and AMPK-dependent processes [52–55]. Metformin’s effect on satellite cells may in part be due to its anti-inflammatory effects. To investigate metformin’s effect on inflammation after severe burn injury, we performed immunohistochemistry and western blotting for NF-κB p65. This protein complex has been implicated in causing muscle wasting in several different diseases [56, 57]. Activation of NF-κB results in the transcription of muscle-specific ubiquitin ligases such as MurF1 that cause protein degradation [58, 59]. Furthermore, NF-κB p65 has been shown to be elevated in the skeletal muscle of mice [37] and in the serum of burn patients [60]. We observed a significant increase in NF-κB p65 protein level and the number of NF-κB p65-positive myonuclei after severe burn injury (Fig. 7), *p* < 0.05. Unlike previous studies, metformin treatment did not attenuate NF-κB p65 activity in this model. It is possible that metformin’s mitigation of muscle atrophy may be attributed to its effect on satellite cell proliferation rather than its anti-inflammatory properties. Further studies are needed to confirm this effect.

## Discussion

Numerous studies have illustrated the protective effect of metformin in mitigating skeletal muscle damage [28–31, 61, 62] and its modulation of stem cell function in the context of injury [24]. Given that metformin is a metabolic drug that can potentially enhance muscle regeneration and stem cell function, we investigated the effect of metformin on the skeletal muscle in response to burn injury. Here, we show that metformin treatment attenuates muscle wasting in response to burn-induced skeletal muscle wasting. Metformin treatment increased gastrocnemius muscle weight and muscle cross-sectional area when compared with the non-treated burn group (Fig. 1b), *p* < 0.05. There was no significant difference between the sham and metformin groups at 7 days indicating a recovery of muscle mass with metformin treatment (Fig. 1b). This rescuing of muscle atrophy is consistent with previous findings showing that metformin rescues muscle wasting in other injury models. Metformin’s attenuation of muscle atrophy after burn injury was also supported by our measurements of the myofiber cross-sectional area (Fig. 1c, d).

To characterize metformin’s mechanisms in mitigating burn-induced muscle wasting, and with the consideration that our latest report revealed temporal changes in Pax7-positive muscle progenitor cells post-thermal injury [37], we examined how satellite cells responded to metformin treatment. Metformin treatment significantly increased the number of muscle progenitors (Pax7^+^) and the protein level of Pax7 when compared with sham and burned animals (Fig. 5), *p* < 0.05. Metformin treatment also significantly increased the proliferation of satellite cells (Fig. 6) as approximately 15% of Pax7+ cells were BrdU positive, *p* < 0.05. In various models of muscle regeneration, a proliferation rate of 15–20% is reported after injury in the skeletal muscle [63–65]. Furthermore, as the severity of muscle injury increases (e.g., polytraumatic injury), the proliferation rate increases to 28% after 1 week post injury [66]. Although the method of inducing muscle injury differs between these studies (e.g., cardiotoxin, cold lesion injury) and our model of burn-induced muscle wasting, they all present almost similar levels of muscle atrophy. As such, the similar proliferation rate araise in our study was expected. Mechanistically, metformin’s metabolic effects might be the underlying mechanism for this pro-proliferatory effect of it. Activated satellite cells proliferate to expand their population and undergo myogenic differentiation into new myofibers [67]. Studies show that stem cells, including satellite cells, rely on glycolysis to provide energy and proliferate [68, 69]. This is due to their deep location within the tissue that limits access to oxygen and protects against damage from reactive oxygen species [70–72]. Satellite cells have few mitochondria and relatively small cytoplasm; hence, they normally have low metabolic rates [73]. When activated, the metabolism of satellite cells rapidly elevates to provide the energy needed for proliferation and differentiation [73]. Like cancer cells, satellite cells rely on Warburg-like glycolysis as the primary source of energy and fast proliferation [50, 74]. As discussed, metformin’s main effect on the skeletal muscle is to increase glucose uptake and glycolysis. Therefore, it is plausible that metformin treatment provides more energy to proliferating satellite cells thus enhancing their ability to regenerate and repair damaged myofibers after burn injury. Recently, Pavlidou et al. reported that metformin reduced the number of Pax7^+^/BrdU^+^ muscle progenitor cells and depleted skeletal muscle regeneration, a finding contrary to ours [75]. The differences between the two studies may be the nature of the injury. We used a burn model to induce muscle wasting rather than cardiotoxin to induce “muscle crush injury.” The local recruitment of inflammatory cells in cardiotoxin injury is different from the systemic inflammatory reaction to burn injury. For example, local muscle injury is characterized by a local increase in neutrophil activity and release of TNF*α* by M1 macrophages which is sustained for up to 2 weeks after injury [76]. Moreover, local cardiotoxin injury specifically increases the expression of osteopontin (OPN), a regulator of muscle inflammation, an event 48 h after injury [77]. Burn injury on the other hand results in a systemic cascade of proinflammatory such as IL-6, TNF, IL-15, MCP-1, and GM-CSF [6]. These cytokines decrease significantly at 2 weeks when there is a switch to anti-inflammatory phenotype [6]. This key difference changes in the nature of the injury between the two studies and may change metformin’s effect on the skeletal muscle. Another difference between the two studies may be the mobility of mice after cardiotoxin injury versus burn injury. Our lab has shown that after severe burn injury, mice are quite mobile [78]. Cardiotoxin injury, however, significantly reduces the mobility of mice post injury [79]. As a result, the differences in mobility will affect the dynamics of muscle proliferation and differentiation, and thus muscle recovery. Lastly, another study showed that metformin protects against cardiotoxin-induced degeneration [31] and metformin’s effects may be context-dependent [80].

To confirm metformin activity within the skeletal muscle after treatment, we performed western blotting for AMPK. AMPK is a master regulator of metabolism which has an α catalytic subunit with two isoforms, α1 and α2 [81]. AMPK’s overall function in the skeletal muscle is to respond to cellular energy deprivation by increasing the potential for ATP production, and AMPK is typically activated during exercise [81]. We observed a significant increase in the protein level of the active form of AMPKα, phospho-AMPKα, in the metformin group after severe burn injury (Fig. 4), *p* < 0.05. This is consistent with the literature showing that metformin exerts its effects through the activation of AMPK in the liver and skeletal muscle [17]. The activation of AMPK in the skeletal muscle after burn injury has important implications in metformin’s observed effect. Recently, AMPK has been shown to be a critical mediator of satellite cell activation and muscle regeneration [50]. In transgenic mice with satellite cell-specific AMPKα1 knockout, there is impairment of the activation and myogenic differentiation of satellite cells during muscle regeneration, thus sustaining muscle atrophy [50]. Researchers also illustrated that activation of AMPK is essential for the Warburg-like glycolysis of satellite cells during muscle regenerations [50]. Therefore, the activation of AMPK we observed in the metformin-treated burn group might be another underlying mechanism to the increase in satellite cell proliferation and attenuate of burn-induced muscle wasting observed (Fig. 4).

Metformin may have also indirectly influenced satellite cell activity through decreasing fat infiltration in the skeletal muscle. After severe burn injury, lipid metabolism is significantly altered resulting in extensive lipolysis [82]. Lipolysis is the breakdown of triacylglycerol into free fatty acids (FFA) and glycerol [82]. The release of free fatty acids contributes to post-burn morbidity and mortality by mediating insulin resistance and increasing fat infiltration in various organs, including the skeletal muscle [82]. Intramuscular fat infiltration is the accumulation of fat within the myofibers themselves [44]. This is because free fatty acids impair insulin-mediated glucose uptake [83, 84] and inhibit glucose transport activity [85]. Furthermore, fat infiltration in the muscle is associated with increased risk of fracture and frailty [86], inflammation [87], and functional deficits [44]. In this animal study, we show that metformin reduces fat infiltration in the skeletal muscle after severe burn injury (Fig. 3). Recent findings from our lab have also shown that metformin reduces fat infiltration in the liver and improves mitochondria bioenergetics. One way by which metformin may reduce fat infiltration is through the activation of AMPK [80]. One of AMPK’s many effects is to inhibit the activity of *Acetyl*-*CoA carboxylase* (*ACC*), a key enzyme in the synthesis of fatty acids [80]. A reduction in ACC activity by metformin treatment may reduce fatty acid synthesis after burn injury leading to a reduction in circulating fatty acids and thus less fat accumulation in organs such as the skeletal muscle and liver. Perhaps this reduction in intramuscular fat infiltration reduces inflammation in the skeletal muscle, thus improving the function of satellite cells and reducing the extent of muscle wasting observed.

Severe burn injury is associated with insulin resistance and hyperglycemia. Clinically, this is detrimental to patients because it is associated with worse outcomes due to increased infections, increased catabolism and hypermetabolism, and increased incidence of pneumonia. The gold standard to treat hyperglycemia is insulin. Insulin treatment achieves tight glucose control and reduces the morbidity of patients. While this is encouraging, there are limitations to insulin treatment. For example, insulin treatment is associated with a fourfold increased risk of hypoglycemia. This is important because patients that experience a hypoglycemic episode have a ninefold increased risk of mortality [82]. Thus, the use of insulin in intensive care units is limited. Alternatively, treating burn patients with an anti-diabetic drug that manages glucose levels with fewer factors than insulin is ideal. Metformin is a drug that can achieve tight glucose control without the added risk of hypoglycemia like insulin. Gore et al. investigated the effect of metformin on severely burn adults through a stable isotope infusion study [39]. One group received metformin treatment (*n* = 8) for 7 days while another received the placebo (*n* = 5) for the duration of the study [39]. In the metformin group, endogenous glucose production decreased by 50%, and serum glucose levels were significantly lower compared to the placebo group [39]. Researchers found that the rate of protein breakdown was unaffected despite the reduction in glucose production and levels [39]. However, there was a net improvement in protein balance in the metformin group due to an elevation in protein synthesis levels [39]. A possible downside to using metformin is the potential for patients to experience lactic acidosis. A randomized phase II clinical trial by Jeschke et al. has demonstrated that metformin decreased glucose levels equally as well as insulin and was safe to use in burn patients. Furthermore, metformin treatment is not associated with lactic acidosis in burn patients [82]. A systemic review of 347 clinical trials found no evidence of fatal lactic acidosis [88].

There are a few limitations to our study which could be addressed in future studies. First, besides in vivo, we can explore the mechanisms of metformin further through in vitro studies. For instance, metformin’s effect on satellite cells can be confirmed by isolating satellite cells from humans or mice and treating them with metformin. Based on our current study, we would expect metformin to increase proliferation of satellite cells in vitro. Moreover, treating satellite cells with metformin and dorsomorphin (a reversible and selective AMPK inhibitor) and examining their effects on proliferation is important. This will help determine whether metformin’s effect on satellite cells is through AMPK or some other mechanisms independent of AMPK. Unfortunately, a challenge with in vitro studies is replicating the inflammatory niche unique to burn injury. Therefore, we believe that the value of in vivo studies is greater. Second, repeating our study with Pax7 reporter mice to trace the lineage of proliferation and differentiation after severe burn injury would be insightful [35]. Furthermore, developing mice that are deficient for AMPK in Pax7 reporter cells is important to uncover the mechanisms of metformin on satellite cells after burn injury. Last, using mice models may not accurately replicate post-burn hypermetabolism observed in burn patients [78]. Larger animal models like porcine would be more reflective of humans [78].

## Conclusion

In summary, our work shows that metformin mitigates burn-induced muscle wasting in vivo through enhancement of a myogenic phenotype by affecting Pax7-positive skeletal muscle progenitor cells. The underlying mechanism might mainly rely on the activation of AMPK, modulation of muscle progenitor activity, or reduction of fat infiltration in the muscle. These findings, in conjunction with recent findings illustrating the safety and efficacy of metformin treatment in burn patients, support the notion that long-term treatment with metformin could have beneficial effects in attenuating hypermetabolism and muscle catabolism in burn patients. Future research should focus on the development of therapies that address burn-induced alterations in satellite cell activity to maximize the recovery of muscle mass in burn patients.

## Supplementary information

**Additional file 1.** Ratio of muscle weight to body weight in sham, burn, burn + metformin at 7 days post-burn-in mice.

**Additional file 2.** Quantification of BrdU^+^/ Pax7^−^ cells in sham, burn, burn + metformin at 7 days post-burn-in mice. B) Quantification of Pax7^+^/ BrdU^−^ cells in sham, burn, burn + metformin at 7 days post-burn-in mice.

## Data Availability

The data generated from this study are available from the corresponding author upon reasonable request.

## Abbreviations

*ACC*: *Acetyl*-*CoA carboxylase*
AMPK: AMP-activated protein kinase
CSA: Cross-sectional area
FFA: Free fatty acids
GLUT4: Glucose transporter type 4
Pax7: Paired-box transcription factor 7
TBSA: Total burn surface area
T2D: Type 2 diabetes
