# Supplementary figures and images for: Metformin alleviates muscle wasting post-thermal injury by increasing Pax7-positive muscle progenitor cells
Source: Stem Cell Res Ther. 2020 Jan 8;11:18. doi: 10.1186/s13287-019-1480-x (PMC6950874; doi:10.1186/s13287-019-1480-x)

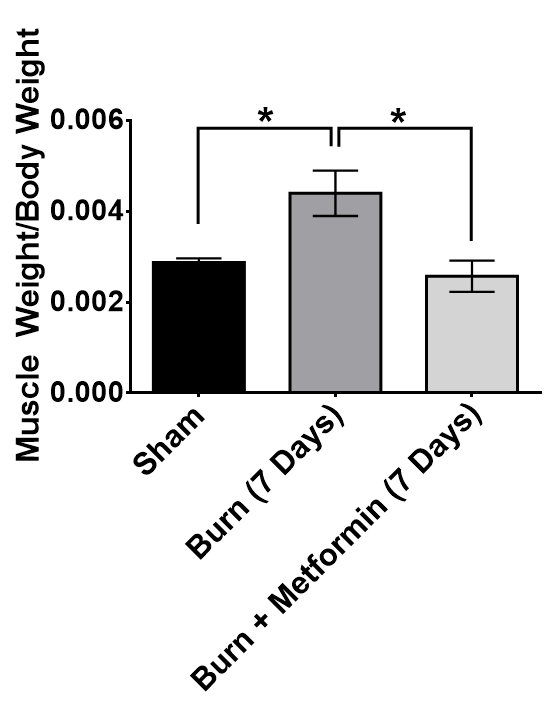

Supplement: Supplementary file 1 — Additional file 1. Ratio of muscle weight to body weight in sham, burn, burn + metformin at 7 days post-burn-in mice. [file 13287_2019_1480_MOESM1_ESM.tif]

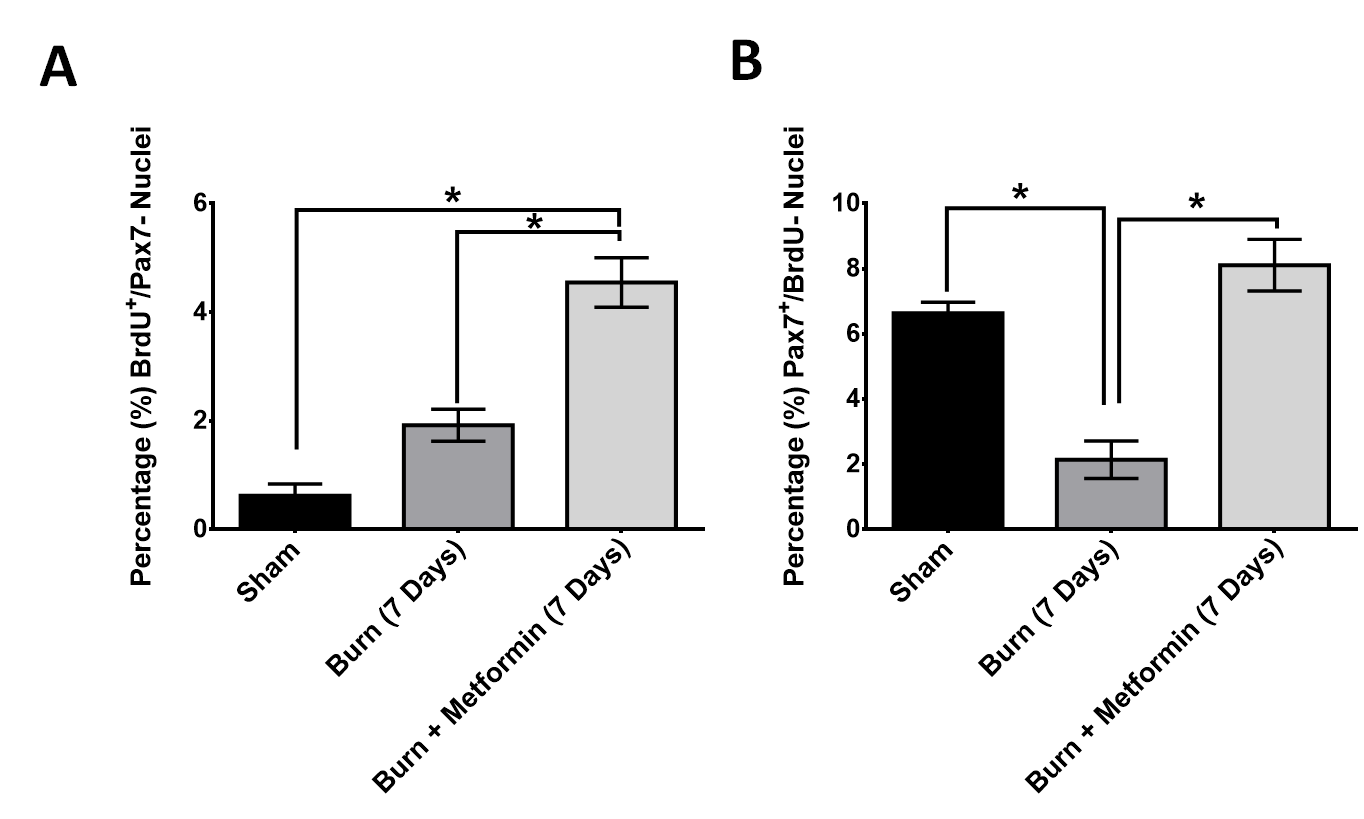

Supplement: Supplementary file 2 — Additional file 2. Quantification of BrdU+/ Pax7− cells in sham, burn, burn + metformin at 7 days post-burn-in mice. B) Quantification of Pax7+/ BrdU− cells in sham, burn, burn + metformin at 7 days post-burn-in mice. [file 13287_2019_1480_MOESM2_ESM.tif]
